# Robotic Surgery for Oropharyngeal Cancer

**DOI:** 10.5041/RMMJ.10148

**Published:** 2014-04-28

**Authors:** Shivani Shah, David Goldenberg

**Affiliations:** Division of Otolaryngology—Head and Neck Surgery, Department of Surgery, The Pennsylvania State University—Milton S. Hershey Medical Center, Hershey, Pennsylvania, USA

**Keywords:** Human papillomavirus, oropharyngeal cancer, transoral robotic surgery

## Abstract

Oropharyngeal cancer represents a growing proportion of head and neck malignancies. This has been associated with the increase in infection of the oropharynx by oncogenic strains of human papillomavirus (HPV). Transoral robotic surgery (TORS) has opened the door for minimally invasive surgery for HPV-related and non-HPV-related oropharyngeal cancer. Compared to traditional open surgical approaches, TORS has been shown to improve functional outcomes in speech and swallowing, while maintaining good oncologic outcomes.

## INTRODUCTION

The oropharynx is the posterior continuation of the oral cavity. It is separated from the nasopharynx superiorly by the soft palate and the hypopharynx inferiorly by the base of the tongue at the level of the hyoid. Anteriorly, the junction of the hard and soft palates represents the border between the oral cavity and the oropharynx. Additional structures within the oropharynx include both lateral and posterior pharyngeal walls, soft palate, bilateral tonsillar regions, and base of tongue. Cancers of the tonsillar region and base of tongue make up the bulk of cases, whereas tumors of the pharyngeal walls and soft palate are much less common.

The overall incidence of oropharyngeal cancer has been steady since the mid-1970s.^1^ Unlike other head and neck malignancies, the incidence has not decreased in association with the decreasing prevalence of one of the major risk factors, cigarette smoking. This discrepancy has been attributed to the increasing proportion of oropharyngeal cancers which are related to human papillomavirus (HPV) infection. These HPV-related tumors occur in younger patients, are more likely to occur in never-smokers and never-drinkers, and have better survival rates than HPV-negative tumors.^2^,^3^

Management of oropharyngeal cancers generally involves a combination of surgery, radiation, and chemotherapy. Historically, locally advanced cancers of the tonsils and tongue base have been difficult to visualize from a transoral viewpoint and required extensive tissue dissections from an open approach or were treated predominantly with chemotherapy or radiation. The introduction of transoral robotic surgery (TORS) has allowed an increase in the ability to manage oropharyngeal cancer via primary minimally invasive surgery.^4^ In the context of a rising number of HPV-related cancers, TORS is an increasingly important tool in the approach to management of oropharyngeal cancer.

In this article, we review the role of transoral robotic surgery (TORS) in the management of oropharyngeal cancers, and specifically how this minimally invasive technique will affect the management of HPV-related tumors.

## HISTORICAL PERSPECTIVES

While robotic technology has been routinely used for industrial purposes for over 60 years, it was not until relatively recently that it was introduced to the field of surgery. The first reported employment of a surgical robot was in 1985 when the PUMA 560 robot was used by a group of neurosurgeons in California to improve the accuracy of CT-guided stereotactic biopsies.^5^ Urologists were not far behind, and within 6 years the same PUMA 560 was used to perform the first minimally invasive robotic procedure during a transurethral resection of the prostate.^6^ From there, robot-assisted procedures continued to develop and became popular in a number of other specialties including gynecologic, cardiothoracic, orthopedic, and general surgery.

In spite of its growing popularity, application of this new technology by otolaryngologists was initially quite limited. The early instruments were designed for use in spacious cavities, such as the abdomen or pelvis, with widely spaced access ports. They were bulky and not well-designed for the anatomic constraints of the head and neck. However, as robot technology continued to adapt for use in surgery and newer instruments were developed, head and neck surgeons began developing transoral robotic surgery (TORS).^7^

In 2005, McLeod and Melder performed the first transoral robotic-assisted procedure when they used the da Vinci surgical robot (Intuitive Surgical, Inc., Sunnyvale, CA, USA) to excise a vallecular cyst.^8^ During that same time, O’Malley et al. were experimenting with transoral base of tongue excisions on cadavers and dogs.^9^ In 2006, they reported the first three live patients to undergo TORS for base of tongue neoplasms in a prospective clinical trial.^10^ From there, research in TORS gained momentum. Two larger studies, with 49 and 54 patients respectively, were published in the next few years suggesting the use of TORS as a feasible and efficacious alternative to traditional operative methods, with good functional outcomes.^11^–^13^ Growing interest in transoral robotic surgery ultimately culminated in the US Food and Drug Administration (FDA) approval of the use of TORS for management of select benign and malignant tumors of the head and neck in 2009.^14^

## CURRENT APPLICATIONS IN OROPHARYNGEAL CANCER

Transoral robotic surgery is currently available at most tertiary medical centers in the United States. It is also actively being adopted at major medical centers in Europe.^15^ At centers where the technology and expertise are available, many oropharyngeal cancers are amenable to transoral robotic resection. Important considerations when deciding on the use of TORS include tumor characteristics, such as deep neck invasion and involvement of major blood vessels, and also anatomical factors such as clinically significant trismus.^11^,^13^ Most transoral robotic oropharyngeal resections are within the tonsillar fossa and tongue base, reflecting the relatively higher clinical prevalence of these tumors compared to soft palate, uvula, and posterior pharyngeal wall neoplasms.

The majority of studies published include both early and advanced-stage cancers. A few studies to date have evaluated TORS specifically for advanced-stage oropharyngeal cancers. In 2010, Weinstein et al. looked prospectively at 47 patients with stage III and IV oropharyngeal cancer treated with primary TORS. Staged neck dissection and adjuvant therapy were included in patient management as clinically indicated. They found that disease-specific survival was 90% at 2 years and comparable to previously published data on chemoradiotherapy studies. They also noted good functional outcomes, including low rates of feeding tube dependence and permanent tracheostomy.^12^

### Tonsillar Fossa

Studies have shown that surgery is highly effective in treating tonsillar cancer and provides accurate staging information for adjuvant therapy;^16^ however, the morbidity of an open surgical approach can be significant. It frequently requires a mandibulectomy, tracheostomy, feeding tube, and long-term speech therapy for dysphagia. Additionally, transoral resection of tonsillar lesions has previously been restricted to tumors that are limited to the tonsillar fossa, with minimal involvement of surrounding structures, due to limited visualization. In 2007, Weinstein et al. described TORS for radical tonsillectomy in 27 patients with invasive squamous cell carcinoma of the tonsil. Their exclusion criteria were limited to: 1) unresectable neck nodes, 2) mandibular invasion, 3) involvement of >50% of the tongue base, 4) involvement of >50% of the posterior pharyngeal wall, 5) carotid artery involvement, or 6) fixation to prevertebral fascia. Only two patients required tracheotomy at any point during the study, and 26 of the 27 patients were able to swallow without difficulty at their last follow-up visit. Twenty-five of the 27 tumors were resected with negative margins, and there were no local or regional recurrences.^17^ This study suggests that TORS for tonsil-based cancers can produce similar oncologic outcomes as other modalities with improved functional results.

Since that initial description of TORS for radical tonsillectomy, other studies have also demonstrated similar favorable oncologic and functional outcomes. In 2009, Moore et al. looked at 45 patients undergoing transoral robotic surgical excision, 19 of which were for tonsillar fossa tumors. Of these, none required tracheostomy tube placement, and one patient with a T4 tumor required percutaneous endoscopic gastrostomy (PEG) tube placement for feeding access. During the relatively short reported follow-up period, they achieved excellent disease control, with only one patient developing a contralateral parapharyngeal metastatic lesion.^11^ Recently, More et al. compared functional swallowing outcomes after TORS with outcomes after primary chemoradiation therapy for stage III and IV tonsillar cancer. They found significantly better scores on the MD Anderson Dysphagia Inventory (MDADI) at 6 and 12 months postoperatively for those patients treated with TORS.^18^

### Base of Tongue

Similar to tonsillar cancers, previous options for surgical management of base of tongue tumors were effective in achieving local control, but did not come without significant morbidity of speech and swallowing. Research suggests that TORS has the potential to achieve good locoregional control of base of tongue cancers while decreasing some of the morbidity. In the previously mentioned Moore et al. study, of the 45 patients with oropharyngeal squamous cell carcinoma who underwent transoral robotic excision, 26 of the cases were base of tongue primary tumors.^11^ Fourteen of these (54%) required tracheostomy for an average length of 7 days before decannulation. Seven patients (27%) with advanced T3 or T4 base of tongue disease required PEG tubes for enteral support due to aspiration. At 4 weeks postoperatively, 90% of all of the patients in the study were able to resume an oral diet.^11^ These functional outcomes are favorable when compared to similar studies of outcomes following an open resection.^19^,^20^ From the oncologic perspective, follow-up was less than 16 months, but only one patient with base of tongue primary tumor had a local recurrence in that limited time period.^11^ Similarly, Mercante et al. also reported favorable outcomes with TORS for base of tongue neoplasms. In a series of 13 patients with T1 and T2 tumors, 12 patients had negative surgical margins. Tracheostomy and nasogastric feeding was required for a mean of 6 and 7.5 days, respectively, and overall patients had good functional outcomes.^21^

Transoral robotic base of tongue resection has been found to be useful in a diagnostic capacity in the setting of unknown primary head and neck malignancy. In 2013, Mehta et al. investigated 10 patients with unknown primary tumors of the head and neck. After imaging, endoscopy, cervical biopsy, and bilateral tonsillectomy, patients underwent TORS for base of tongue resection. Nine of the 10 patients had successfully identified base of tongue primary lesions following the resection. Of these, one patient actually did not require any adjuvant therapy as the primary tumor had been completely resected. Postsurgical functional outcomes were again promising, with nine of the 10 patients tolerating soft diet at first follow-up and only one patient requiring PEG tube placement.^22^

## ADVANTAGES

The advantages of using TORS to manage oropharyngeal cancers are multifocal with regard to oncologic, technical, and functional outcomes. First, primary surgical excision with TORS, as opposed to primary chemoradiation, allows the tumors to be accurately staged. It has been found that surgical staging alters clinical staging in 40% of cases, which subsequently can affect further management and the need for adjuvant therapy.^23^

Second, there are technical benefits to operating with a robot. The cameras allow visualization of an anatomic location that is typically poorly visualized using headlamps and mirrors. The operating field is visualized in three dimensions with 10-fold magnification. The robotic arms also filter tremors, allowing precision with microscopic movements. Compared to endoscopic tools, the robotic instruments also have more freedom of articulation and eliminate the “fulcrum effect.”^24^ These factors contribute to the third advantage, which is improved postoperative oropharyngeal function. TORS enables preservation of the maximum amount of healthy muscle and neurovascular tissue. Markers of long-term function, including tracheostomy tube and gastrostomy tube dependence, have been shown to be as low as 1.5% and 4.5%, respectively, 2 years after TORS for resection of oropharyngeal cancer.^25^

## DISADVANTAGES

The transition to TORS for oropharyngeal cancer management is not without disadvantages. Although sometimes overlooked, cost is a critical factor in robotic surgery. Estimates of buying and installing one robotic system fall between 1 million and 2.5 million US dollars.^26^,^27^ This does not include ongoing costs of maintenance and instrument replacement. These costs are in turn transferred to the patients who are already facing an expensive disease.

**Figure 1.**
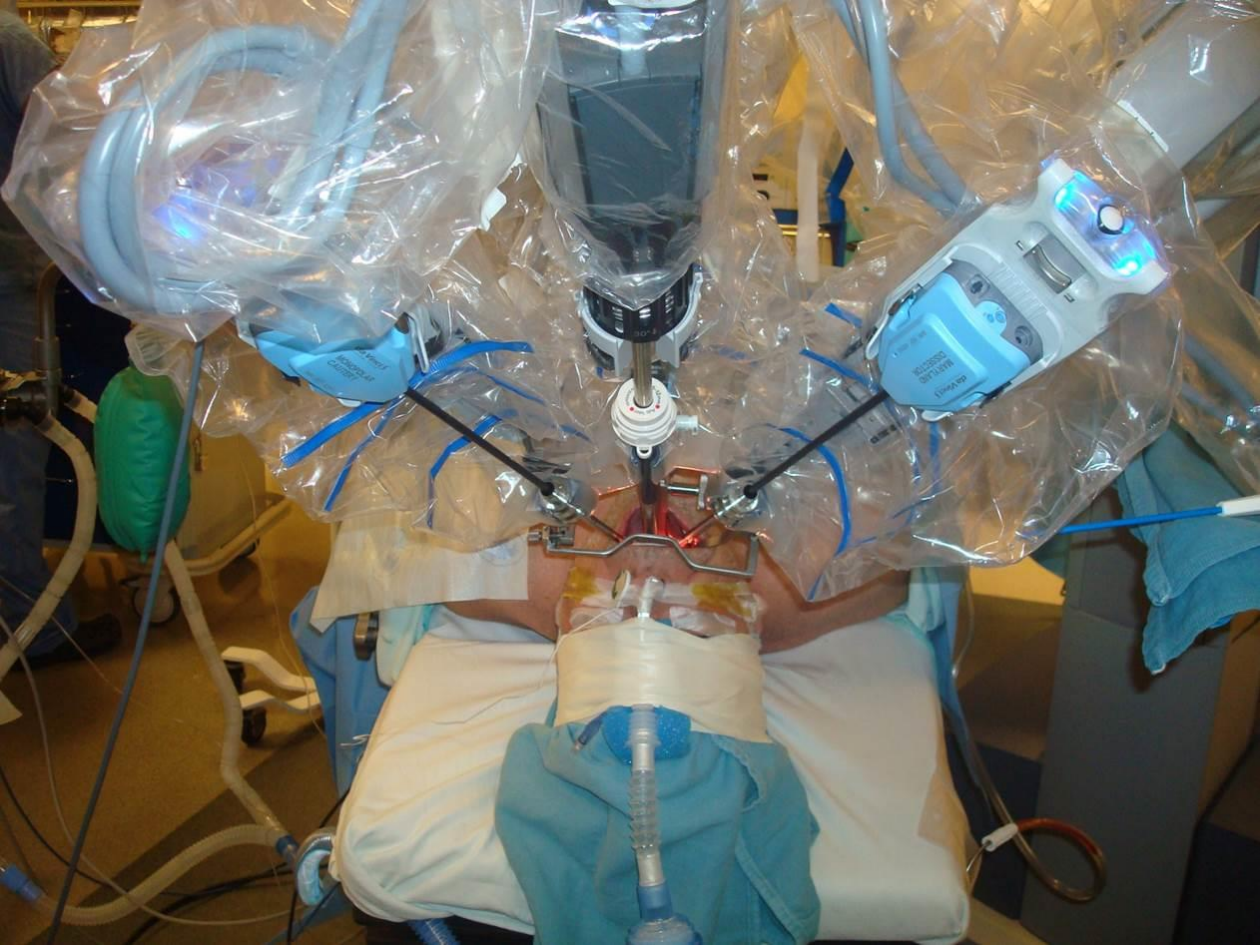
Robotic Instrumentation Set-up during Transoral Robotic Procedure.

In addition, from a surgical perspective, robots are not well-designed for use in the oropharynx. The bulky instruments are predominantly designed for use in the abdominal and pelvic cavities and can be cumbersome within the limitations of the oral cavity. The added number of instruments required also greatly increases the complexity of and time for operating room set-up (Figure 1). With extensive instrumentation near the face and eyes, concerns have also been raised regarding patient safety. Hockstein et al. performed a cadaveric study, early in the development of TORS, examining the safety profile of robotic instrumentation as compared to traditional transoral tools and found that few additional risks were accrued by using the robot.^26^ However, this technical question about TORS still requires more time and investigation.

Experienced surgeons also comment that the lack of tactile feedback is an important concern when using robotic instruments and can lead to mishandling of delicate tissues.^24^ This contributes to the significant learning curve associated with utilizing the robot. Length of time that a patient is intubated, operative time, and technical complications such as bleeding have been shown to be increased early in a surgeon’s learning curve with TORS. However, these factors decrease significantly with surgeon experience.^28^ Consequently, reported outcomes for TORS may unfavorably vary from actual outcomes in certain circumstances. It is important to consider some of these factors before adopting TORS in practice.

## TORS FOR HPV-RELATED CANCERS

Oropharyngeal cancer that is related to HPV infection differs from non-HPV-related oropharyngeal cancer in a number of ways. Patients affected by HPV-related cancers are typically younger at diagnosis and also more likely to be never-smokers and never-drinkers. Three-year survival rates have also been shown to be better for HPV-related cancers (82% versus 57% in HPV-negative patients).^2^ As such, it is important to consider that optimal management of HPV-related tumors may also need to be different from non-HPV-related tumors. More specifically, these younger patients with improved prognoses may be good candidates for minimally invasive, function-sparing techniques such as TORS.

In 2010, Cohen and colleagues established that despite differences in prognosis and outcomes between HPV-positive and HPV-negative oropharyngeal cancers, TORS is effective as a primary treatment modality in both subsets of patients. In their review of 50 patients with oropharyngeal cancer managed with primary TORS, there was no statistically significant difference in disease-specific survival based on HPV status.^29^

On the other hand, some studies have suggested that HPV status has a significant impact on the effectiveness of TORS in treating oropharyngeal cancer. It has been suggested that TORS alone, without adjuvant therapy, may be adequate treatment for HPV-positive oropharyngeal cancer. Recently, Olsen et al. reported a study of 18 patients with T1–T3 oropharyngeal tumors with N0–N2a neck disease who underwent surgery alone (TORS with neck dissection) and no adjunct therapy. Twelve of the 18 patients were non-smokers with HPV-positive tumors and, in this specific subpopulation, 3-year survival was 100%, with 91% recurrence-free survival. Only 3 of 18 required tracheostomy tube placement, and no patients required gastrostomy tube for enteral support.^30^ This suggests that, in a subset of non-smoking patients with HPV-related oropharyngeal cancer, excellent oncologic and functional outcomes are possible with TORS and neck dissection alone.

More specific than HPV status, the latest studies looking into the prognosis of oropharyngeal carcinomas are examining the expression of the protein p16^INK4a^. The expression of p16^INK4a^ is variable amongst oropharyngeal tumors, but has a strong association with HPV positivity. In a study published within the last year, Quon et al. reported that p16^INK4a^ expression has not yet been associated with any significant difference in treatment outcomes, but limitations to this study leave room for further investigation.^31^

## FUTURE DIRECTIONS

Currently, transoral robotic surgery may still be considered to be in its infancy. Only 4 years have passed since FDA approval for its use in head and neck tumors. There are ongoing advances in robot technology, including those specific to head and neck surgery. New instruments are being developed that are smaller and better adapted for use in the oral cavity.

In addition, more studies are being done on the indications for TORS as well as the different outcomes. Comparison studies of TORS versus other treatment modalities in the management of oropharyngeal cancers are still needed. Also, not enough time has passed for sufficient research into long-term outcomes of TORS beyond 5 to 10 years. These are all areas of current ongoing research that have the potential greatly to affect the future direction of TORS.

## CONCLUSIONS

Transoral robotic surgery is a quickly developing technique in the management of oropharyngeal cancers. Studies of both tonsillar and tongue base tumors have shown favorable functional and oncologic outcomes after TORS. However, more research is still needed to evaluate long-term outcomes beyond 5–10 years. In the context of a growing proportion of HPV-related oropharyngeal cancers, TORS provides patients with a minimally invasive treatment option. Given the improved prognosis of this subset of oropharyngeal cancers, they may be amenable to single modality treatment. TORS alone has shown promising results in treating HPV-positive tumors. There are no head-to-head studies comparing TORS alone to chemoradiation alone in the management of HPV-positive cancers, and this will likely be an area of future study.

## Abbreviations:

FDA: Food and Drug Administration
HPV: human papillomavirus
PEG: percutaneous endoscopic gastrostomy
TORS: transoral robotic surgery.
